# Acetylation Increases EWS-FLI1 DNA Binding and Transcriptional Activity

**DOI:** 10.3389/fonc.2012.00107

**Published:** 2012-09-07

**Authors:** Silke Schlottmann, Hayriye V. Erkizan, Julie S. Barber-Rotenberg, Chad Knights, Amrita Cheema, Aykut Üren, Maria L. Avantaggiati, Jeffrey A. Toretsky

**Affiliations:** ^1^Lombardi Comprehensive Cancer Center, Georgetown UniversityWashington, DC, USA

**Keywords:** EWS-FLI1, acetylation, Ewing’s sarcoma, PCAF

## Abstract

Ewing Sarcoma (ES) is associated with a balanced chromosomal translocation that in most cases leads to the expression of the oncogenic fusion protein and transcription factor EWS-FLI1. EWS-FLI1 has been shown to be crucial for ES cell survival and tumor growth. However, its regulation is still enigmatic. To date, no functionally significant post-translational modifications of EWS-FLI1 have been shown. Since ES are sensitive to histone deacetylase inhibitors (HDI), and these inhibitors are advancing in clinical trials, we sought to identify if EWS-FLI1 is directly acetylated. We convincingly show acetylation of the C-terminal FLI1 (FLI1-CTD) domain, which is the DNA binding domain of EWS-FLI1. *In vitro* acetylation studies showed that acetylated FLI1-CTD has higher DNA binding activity than the non-acetylated protein. Over-expression of PCAF or treatment with HDI increased the transcriptional activity of EWS-FLI1, when co-expressed in Cos7 cells. However, our data that evaluates the acetylation of full-length EWS-FLI1 in ES cells remains unclear, despite creating acetylation specific antibodies to four potential acetylation sites. We conclude that EWS-FLI1 may either gain access to chromatin as a result of histone acetylation or undergo regulation by direct acetylation. These data should be considered when patients are treated with HDAC inhibitors. Further investigation of this phenomenon will reveal if this potential acetylation has an impact on tumor response.

## Introduction

Ewing sarcoma (ES) is the second most frequent primary bone tumor of childhood and early adolescence. It is associated with specific chromosomal translocations, resulting in an in-frame fusion of the 5′ transactivation domain of EWS to the 3′ DNA binding domain of an ETS family transcription factor. *FLI1*, *ERG*, *ETV1*, *ETV4*, and *FEV* have all been described as fusion partners for *EWS* in ES (Khoury, 2005). The EWS-ETS fusion is considered causative in the development of ESs since the aberrant transcription factors deregulate the cellular gene expression program (Hancock and Lessnick, 2008). Patients with ES require new therapy that both decreases mortality and reduces long term morbidity (Esiashvili et al., 2008). EWS-*ets* are recognized therapeutic targets, yet little is known about their post-translational regulation.

During the past decade, acetylation has emerged as a key mechanism for post-translational regulation of both histones and transcription factors like NF-kB, p53, GATA, and many others (Spange et al., 2009). Acetylation is a reversible modification, in which histone acetyltransferases (HAT) transfer the acetyl moiety from acetyl-CoA to the ε-amino groups of lysine residues and is reversed by histone deacetylases. Acetylation by nuclear A-type HATs is directly linked to transcription regulation (Spange et al., 2009) and are subdivided into five families: GNAT (Gcn5-related), MYST (e.g., TIP60), p300/CBP, basal/general transcription factors, and nuclear receptor co-factors. They do not acetylate lysine moieties randomly but instead often use the motif GKxxP, where the acetylated lysine is preceded by a glycine. New sites are being rapidly discovered (Choudhary et al., 2009; Smith and Workman, 2009) showing that this motif has serious limitations in predicting non-histone protein acetylation. Numerous HATs furthermore undergo functionally relevant auto-acetylation (Thompson et al., 2004). The consequences of acetylation include alterations in protein stability, protein–protein interaction, DNA binding, and transcription activation. Histone deacetylase inhibitors (HDI) are advancing in clinical trials (Tan et al., 2010); thus enhanced knowledge of the effects of acetylation can inform therapeutic trials.

Little is known about post-translational modification of the EWS-FLI1 fusion protein, and there are no reports describing lysine acetylation (Klevernic et al., 2009). EWS-FLI1, EWS-ER81, and EWS-ATF form complexes with the acetyltransferases p300 (Fuchs et al., 2003) and CBP (Fujimura et al., 2001; Araya et al., 2003) leading to transcriptional activation. When EWS-FLI1 interacts with p300 alterations in histone acetylation are observed (Nakatani et al., 2003). When ES cells are treated with HDI, EWS-FLI1 protein and mRNA levels decrease, however acetylation of EWS-FLI1 was not reported (Sakimura et al., 2005). Studies with the HDI MS-275 showed an average IC_50_ in the nanomolar range (100 nM to 1 μM) in ES cells accompanied by de-repression of the EWS-FLI1 target TGFβRII, re-expression of the histone acetylation sensitive locus p21 and a dose-dependent decrease in tumor volume in MS-275-treated mice (Jaboin et al., 2002). The HDI vorinostat recently completed Phase I testing for childhood cancer (Fouladi et al., 2010).

The therapeutic utility of HDI would increase dramatically if critical acetylation targets were identified. We provide evidence that the co-expression of EWS-FLI1 with histone acetylases increases EWS-FLI1 transcriptional activity based upon increased binding to DNA. The C-terminal region of EWS-FLI1 was fully characterized *in vitro*, for acetylation marks. However, the ability to identify these marks on full-length EWS-FLI1 either *in vitro* or *in cell based assays* was not successful. These data provide a mechanistic insight into EWS-FLI1 function which may potentially lead to pharmacodynamic models of inhibitor activity.

## Experimental Procedures

### Cell culture

Ewing sarcoma cells (A4573, EWS-FLI1 type III; SKES-1, EWS-FLI1 type II; TC32 and TC71, EWS-FLI1 type I) were maintained in RPMI, 10% FBS under standard cell culture conditions. Cos7 cells were maintained in DMEM, 10% FBS. HDI treatment was carried out with 2 μM suberoylanilide hydroxamic acid (SAHA, also known as vorinostat) or 0.3 μM Trichostatin A (TSA) for 8 and 16 h, respectively.

### Western blotting, antibodies

Western blots were carried out as previously described (Beauchamp et al., 2009). Antibodies used are: α-HA-Tag (6E2, Cell Signaling); α-Flag and α-Flag beads (Sigma); a-acetyl-Lysine [Upstate, 4G12, 06-933 (mouse); Stressgen, KAP-TF120 (rabbit)], α-FLI1 (C-19, sc-356), α-PCAF (E-8, sc-13124), α-CBP (A-22, sc-369), α-p300 (N-15, sc-584) all from Santa Cruz; α-PARP (C2-10; Trevigen). Acetylation site-specific acetyl-EWS-FLI1 antibodies using the following peptides: K240Ac (NH2−) CMNSGLN(K-Ac)SPPLGG (−CONH2), K252Ac (NH2−) CGAQTIS (K-Ac)NTEQRP (−CONH2), K380Ac (NH2−) CTESSMY(K-Ac)YPSDIS (CONH2), K397Ac (NH2−)CYHAHQQ(K-Ac)VNFVPP (CONH2) were made in cooperation with Innovagen (Sweden). Their specificity was tested as shown in Figure 6 by competition experiments using acetylated peptides.

### Transfection, DNA constructs, and immunoprecipitation

Cos7 cells were transfected using Fugene6 (Roche), ES cells were electroporated with the Cellporator (Life technologies). DNA constructs used are pCINeo-EWS-FLI1 pcDNA4TO-PCAF, pcDNA4TO-PCAFΔHAT, and p300-CHA as well as the corresponding empty vectors. For reporter assays the EWS-FLI1 responsive NR0B1 microsatellite luciferase construct was used, kindly provided by Dr. Steven Lessnick (University of Utah; Gangwal et al., 2008). Generally cells were lysed ∼30 h post transfection. Immunoprecipitations were carried out for 4 h at 4°C using Protein G agarose beads (Invitrogen).

### Reporter assay

For reporter assays Cos7 cells were plated the day before transfection and transfected in triplicates. Cells were lysed in passive lysis buffer (Promega) 30–36 h post transfection and subjected to luciferase measurement using Dual-Luciferase-Reporter Assay System (Promega). Treatment of reporter assays with 0.3 μM TSA or 2 μM SAHA was carried out for 8 h prior to cell harvest.

### Inclusion body and protein purification

Recombinant proteins were expressed as 6×His tagged proteins in BL21 bacteria. Inclusion bodies were isolated using *BugBuster* protein extraction reagent (Novagen) according to the manufacturer’s protocol. Inclusion bodies were subsequently solubilized in binding buffer (see below), filtered (0.2 μm), and subjected to column purification (1 ml HiTrap purification columns, ActaPrime system, Amersham) using a refolding method recommended by the manufacturer with the following buffers: refolding buffer (20 mM Tris pH 8, 0.5 M NaCl, 5 mM imidazole), solubilization buffer (refolding buffer plus 8 M urea), binding buffer (refolding buffer plus 6 M guanidine hydrochloride), elution buffer (refolding buffer plus 2 M imidazole). Purified FLI1-C-terminal protein domain (FLI-CTD) was dialyzed (20 mM Tris pH 8.0, 20% glycerol, 100 mM KCl, 0.2 mM EDTA, 1 mM DTT), aliquoted and stored at −80°C. EWS-FLI1 preparations were used as eluted; due to its N-terminal disordered EWS-portion the physical properties of EWS-FLI1 cannot undergo either dialyzing or freezing.

### *In vitro* acetylation

An *in vitro* acetylation reaction with recombinant FLI-CTD contained 50 mM Tris-HCl, pH 8.0, 10% glycerol, 0.1 mM EDTA, 1 mM DTT, 5 mM sodium butyrate, 120 nCi of [^14^C]-acetyl-CoA (55 mci/mmol, Amersham), up to 10 μg of purified substrate protein, and 1.25 μg of recombinant CBP (HAT domain, Biomol) or baculovirus expressed PCAF. Samples were incubated at 30°C for 90 min, and then subjected to SDS PAGE. Gels were fixed, rinsed in enhancer solution, dried, and subjected to autoradiography using Kodak BioMax MS films. For mass spectrometric analysis and EMSA experiments 45 μl non-radioactive acetylation reactions were carried out with up to 30 μg recombinant substrate protein, 1.1 mM Acetyl-CoA (tri-lithium salt, Roche), and 6.5 μg CBP/p300 at 30°C for 90 min. Successful acetylation was verified by western blot analysis using an acetyl-lysine specific antibody (Stressgen). *In vitro* acetylations with the full-length EWS-FLI1 were carried out with smaller protein amounts (∼2 μg) and higher reaction volumes so that the imidazole concentration in the reaction was diluted to <100 mM. Background was subtracted from all signals [as determined with the Multigauge software (Fuji), Q-B/pixel2], then the negative control (samples without enzyme) for each individual substrate was subtracted to give rise to the shown delta (Q-B/pixel2) values.

### Non-radioactive EMSA

For non-radioactive gel-shift assays 1 μg recombinant protein was incubated with 500 fmol of biotin labeled double-stranded oligonucleotides containing the consensus ets-binding site GGAA as previously described (Uren et al., 2004). Complexes were separated on a 6% gel retardation gel (Invitrogen) in 0.5× TBE, and subsequently transferred on Zeta GT (Biorad) membrane. Dried membranes were UV cross-linked for 15 min and subsequently blocked with 10× blocking solution (3.65 g NaCl, 1.2 g Na_2_HPO_4_, 0.5 g NaH_2_PO_4,_ 25 g SDS, dH_2_O to 0.5 L) for 10 min. The biotin labeled probe was detected with horseradish peroxidase labeled streptavidin in a FujiFilm LAS3000 image analyzer.

### Mass spectrometry

Trypsin generated peptides of *in vitro* acetylation reactions were analyzed using ESI LC MS/MS (QStarELITE/TEMPO MDLC system) mass spectrometry (MS) using the software Protein Pilot V3.0 and the UniProt database.

## Results

### EWS-FLI1 mediated transcription increases with acetylation

**Figure 1:**
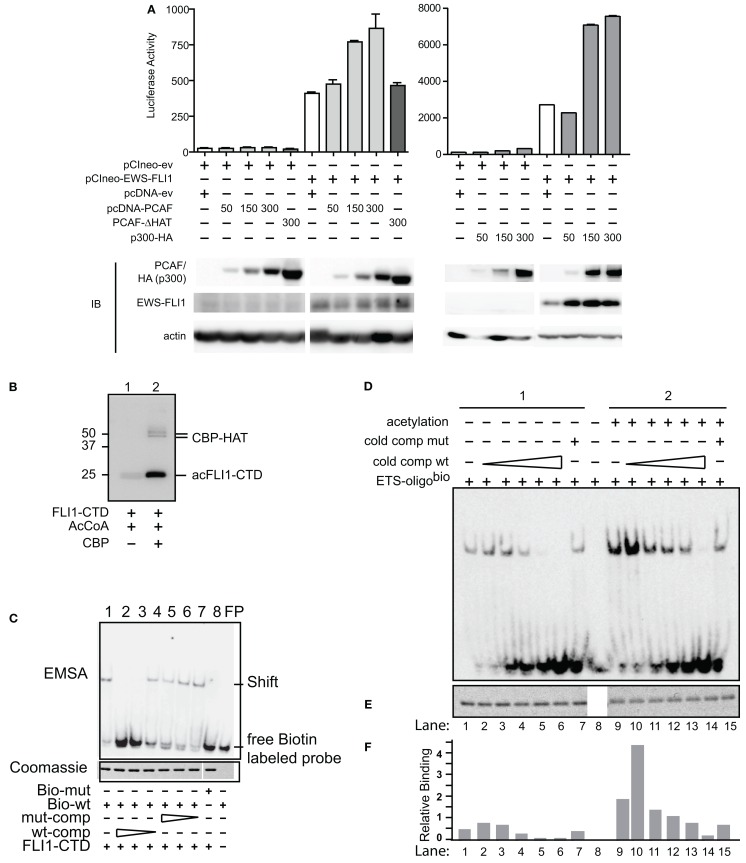
**Acetylases increase transcriptional and DNA binding activity of EWS-FLI1**. **(A)** The EWS-FLI1 responsive NR0B1 promoter (Gangwal et al., 2008) and pCI-EWS-FLI1 were co-transfected into Cos7 cells without PCAF nor p300 (white bar) or with increasing amounts of either PCAF or p300 (gray bars) or a mutant HAT construct lacking the histone acetyltransferase domain (PCAF ΔHAT, black bar). Twenty-four hours later promoter derived luciferase activity as well as protein levels were analyzed. Bar graphs result from triplicate transfection and duplicate luciferase measurement. EWS-FLI1 levels did not exhibit any patterns of change across multiple experiments and those changes here were not representative and thus not considered in final conclusions. **(B)** *In vitro* acetylation was carried out as described and samples of the reaction were tested for successful acetylation by western blot using an anti-acetyl-lysine antibody. **(C)** Recombinant, refolded FLI1-CTD specifically binds to the ets-binding site of dsDNA. The specificity of the FLI1-CTD for the ets-binding site in the oligonucleotide was proven by the lack of binding to mutant oligonucleotide (lane 8), by the competition of unlabeled wild type oligonucleotide (lanes 2–4), and by the lack of competition with mutant oligonucleotide (lanes 5–7); comp, competitor; bio, biotinylated. **(D)** Samples were subjected to non-radioactive EMSA using biotin labeled ets binding site as a probe. Unlabeled wild type competitor was used at 1, 2.5, 5, 10, and 50 times excess. Mutant competitor was only used at the highest concentration (50 times excess). **(E)** Aliquots of the reactions were tested for equal loading by Coomassie staining. **(F)** Densitometric analysis of **(B,C)**. Relative binding = EMSA signal/Coomassie signal.

Since HDI are potential adjuvant treatment options for patients with pediatric sarcoma, our goal was to evaluate the effects of acetylation upon EWS-FLI1 as a therapeutic target. First we investigated whether HAT coactivate EWS-FLI1 mediated transcription. The EWS-FLI1 responsive reporter construct (NR0B1, Gangwal et al., 2008) and an EWS-FLI1 expression construct were transfected into Cos7 cells, along with increasing amounts of a PCAF expression construct (Figure 1A). PCAF enhanced the EWS-FLI1 mediated activation of the luciferase reporter in a concentration dependent manner. However, in the absence of EWS-FLI1, PCAF did not alter the promoter activity. To further support the role of acetylation as a potential cause of the increased activity, a HAT deletion mutant (ΔHAT) of PCAF was used; this acetylase deficient PCAF did not increase the EWS-FLI1 driven reporter activity (Figure 1A, left panel).

In a similar manner as PCAF, expression of p300 along with EWS-FLI1 increased transcription of the NR0B1 promoter (Figure 1A, right panel). Overall, we measured dose-dependent increases in EWS-FLI1 transcriptional activity upon the expression of either HAT. Since the reporter construct lacks histone wrapping, this result was followed by experiments to evaluate whether the increased promoter activity could be due to enhanced EWS-FLI1 binding to DNA secondary to acetylation.

### Acetylation increases DNA binding

EWS-FLI1 DNA binding is well characterized and occurs throughout the *ets* family DNA binding domain of FLI1. For non-radioactive DNA binding assays and MS following *in vitro* acetylation reactions big scale purified full-length EWS-FLI1 preparations were needed. However, this kind of purification in appropriate buffers for acetylation was challenging. Since most of the acetylatable lysine residues are contained in the FLI1 C-terminal domain (CTD), we used this domain in DNA binding studies. We measured FLI1-CTD DNA binding using an electrophoretic mobility shift assay (EMSA). FLIl-CTD was acetylated by the enzymatic domain of CBP (CBP-HAT, Figure 1B). An *ets* binding site oligonucleotide with unlabeled competitor along with mutant sequences were used to assess binding of the FLI1-CTD, followed by quantification of band density (Uren et al., 2004). We evaluated the specificity of the binding using mutant and cold competition (Figure 1C). The acetylated FLI1-CTD demonstrated a four-fold increase in binding compared with non-acetylated control protein (Figures 1D,F, compare column 1 and 9). When titrating in the cold wild-type competitor, a consistent enhancement of acetyl-FLI-CTD binding was measured. Even when 50-fold excess of a mutant competitor was used (matching the highest concentration of the wild-type competitor) the acetyl-FLI-CTD binding was twice as high as the non-acetylated protein (Figures 1D,F, lanes 7 and 15). All gel-shift lanes had equal amounts of protein (Figure 1E). This data shows enhanced DNA binding of acetylated FLI1-CTD which led us to investigate which of the known acetylases might be responsible for EWS-FLI1 acetylation.

### P300 and PCAF can both acetylate recombinant EWS-FLI1

We evaluated each of the three major acetyltransferases independently for their ability to directly acetylate recombinant proteins: full-length EWS-FLI1 (type I 55.4 kDa, 13 lysines), full-length FLI1 (50.9 kDa, 24 lysines), full-length EWS (68.5 kDa, 18 lysines), and the C-terminal FLI1 domain (FLI-CTD, 24 kDa, 12 lysines) were used as substrate. We used full-length p300, PCAF, and the CBP-HAT domain for *in vitro* acetylation assays.

**Figure 2:**
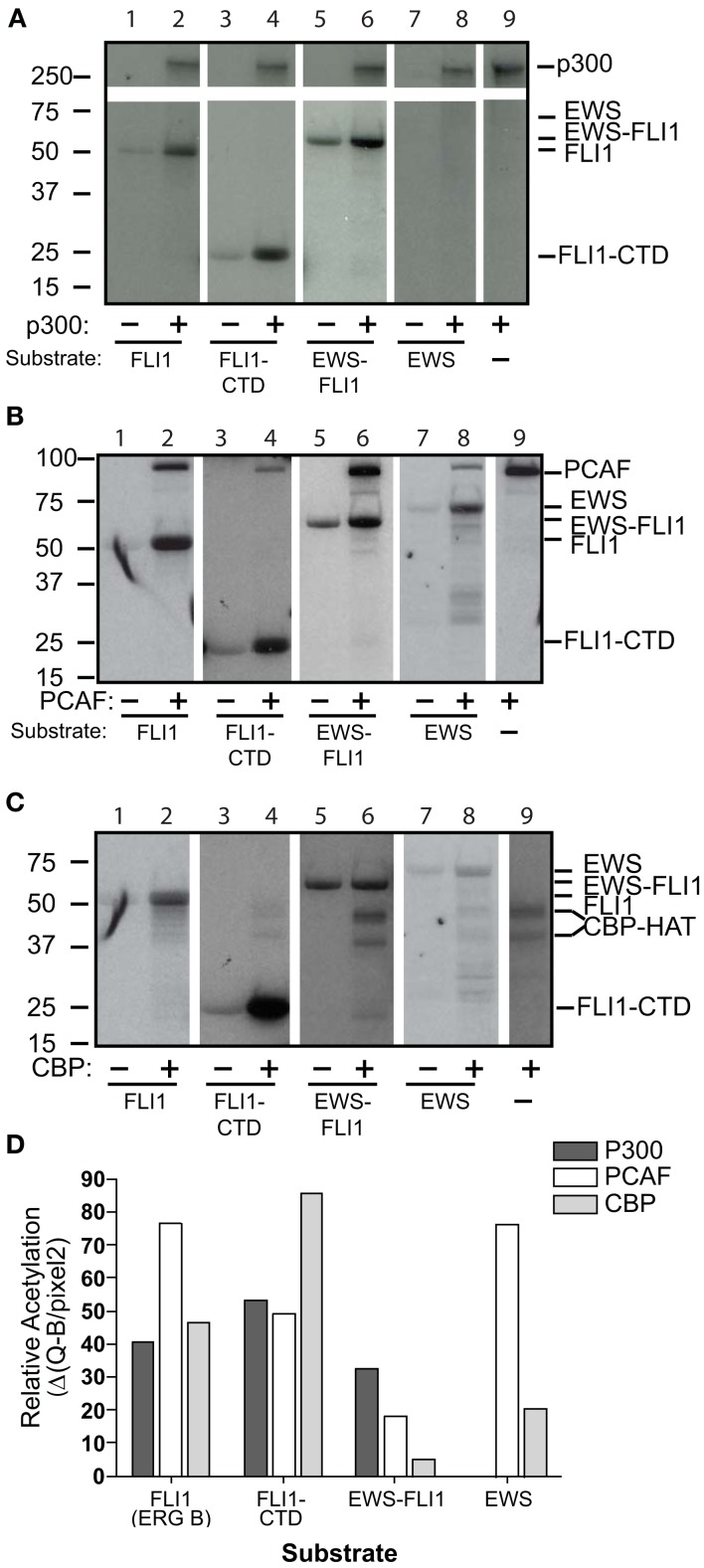
**Full-length EWS-FLI1, and its C-terminal FLI1 domain (FLI1-CTD) become acetylated *in vitro* by p300/CBP and PCAF**. Recombinant, refolded full-length FLI1 (ERG B), FLI-CTD, EWS-FLI1, and EWS were subjected to *in vitro* acetylation (C^14^-AcetylCoA), PAGE, and autoradiography using p300 **(A)**, PCAF **(B)**, and CBP **(C)**. Autoacetylation of the acetyltransferases serves as internal positive control. **(D)** Densitometric analysis of the band intensities shown in **A–C** using the densitometric values/area (Q/pixel2). These acetylations were replications were: the full length EWS and FLI1, EWS-FLI1 each performed twice, the FLI-CTD greater than 10 replicates.

Full-length wild-type FLI1 as well as the FLI1-CTD were strongly acetylated by all three HATs (Figures 2A–C, lanes 1–4). EWS-FLI1 type I was acetylated, but to a lesser degree than FLI1, by both p300 and PCAF, while CBP failed to acetylate the fusion protein (Figure 2, lanes 5 and 6). Full-length EWS was acetylated by both CBP and PCAF while p300 failed to acetylate it (Figure 2, lanes 7 and 8). We addressed the possibility that the non-enzymatic background acetylation of EWS-FLI1 (Figure 2, lanes 5 compared to 6) might result from an unknown acetyltransferase activity of EWS-FLI1 itself. However, our experiments confirmed that EWS-FLI1 has no such enzymatic activity (data not shown). We used densitometric quantification of the band intensity to compare the lanes with the HAT present relative to its absence for each of the different enzymes (Figure 2D).

### Specific lysine residues are identified as the FLI1-CTD acetylation sites by mass spectroscopy

**Figure 3:**
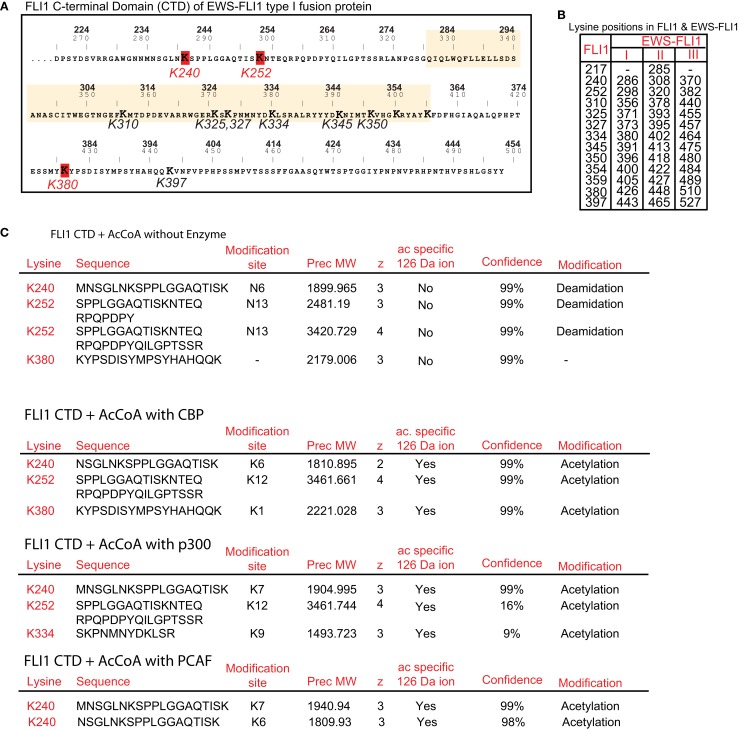
**Identification of individual acetylation sites in FLI1-CTD by mass spectrometry**. **(A)** Sequence of the C-terminal FLI1 domain (FLI1-CTD) as present in all EWS-FLI1 fusion types. Top numbers (BOLD) correspond to amino acid positions in the wild type FLI1 protein; lower numbers refer to positions in the EWS-FLI1 (type I) fusion protein. Lysine residues identified as being acetylated are marked in red and their numbering corresponds to the wild type FLI1 sequence. The ets-domain is boxed in yellow. **(B)** Lysine positions in the full-length wild type FLI1 and the corresponding lysine positions in the three different EWS-FLI1 fusion proteins. **(C)** Results of MS analysis of *in vitro* acetylated FLI1-CTD protein. Recombinant, refolded FLI-CTD was subjected to *in vitro* acetylation by CBP and p300 (no enzyme as control). Trypsin generated peptides were analyzed using ESI LC MS/MS (QStarELITE/TEMPO MDLC system) MS as described in the Section “Experimental Procedures.” The lysine positions most abundantly found as being acetylated are listed.

We sought to identify individual acetylated residues within EWS-FLI1 by means of MS analysis. Twelve of 13 lysine residues of EWS-FLI1 are located in the FLI-CTD (Figure 3A), while the N-terminal EWS-domain attributes only one lysine. Isobaric interference can lead to false assignment of modifications to peptides as acetylation and trimethylation both generate the same +42 kDa mass shift. However, Zhang et al. (2004) demonstrated that a lysine in a peptide modified by methylation or acetylation can be differentiated by MS despite isobaric interference when all of the three following mass spectrometric parameters are used: (1) the acetyl-group characteristic +42 Da mass shift of the b- and y-ion signals of the lysine residues in question, (2) a specific acetyl-lysine marker ion at *m*/*z* 126.09 (126.1), and (3) the acetyl-lysine immonium ion at *m*/*z* 143.1.

We repeatedly detected CBP-dependent acetylation of the lysine residues at positions 240, 252, and 380 (numbering refers to the full-length FLI1 protein, see Figure 3B for amino acid positions in the EWS-FLI1 fusion proteins). Exemplary peptides of the detected acetylation sites are shown in Figure 3C. The same peptides were obtained from non-acetylated and acetylated samples. However, acetylation specific leader ions at 126.09 were detected only in acetylated peptides. Acetylation of lysines K334, K345, K350, and K397 were detected occasionally with CBP (data not shown). Since the ets domain contains an accumulation of lysine residues (which all represent trypsin cleavage sites) most of the generated peptides of the ets domain are too small for efficient resolution by MS. As a result, the DNA binding domain was not well covered by the trypsin generated peptides (77% coverage, data not shown). Therefore, to evaluate acetylation in this region we also generated peptides using the endopeptidase Lys-C (data not shown). Lys-C cleaves at lysine residues but omits the site when the lysine residue is acetylated. Lys-C digestion did not increase the detection of additional DNA binding domain peptides, supportive of the conclusion that Lys residues in the DNA binding domain are not acetylated. As shown above the homolog p300 seemed to be more active in acetylating EWS-FLI1 than CBP. Therefore, we confirmed the MS analysis of the CTD using p300 and detected a similar acetylation pattern (K240 and K252, Figure 3C). In addition we carried out MS analysis with PCAF and obtained acetylation at the same position K240 (data not shown).

### K240, K252, and K380 are validated as acetylation sites

**Figure 4:**
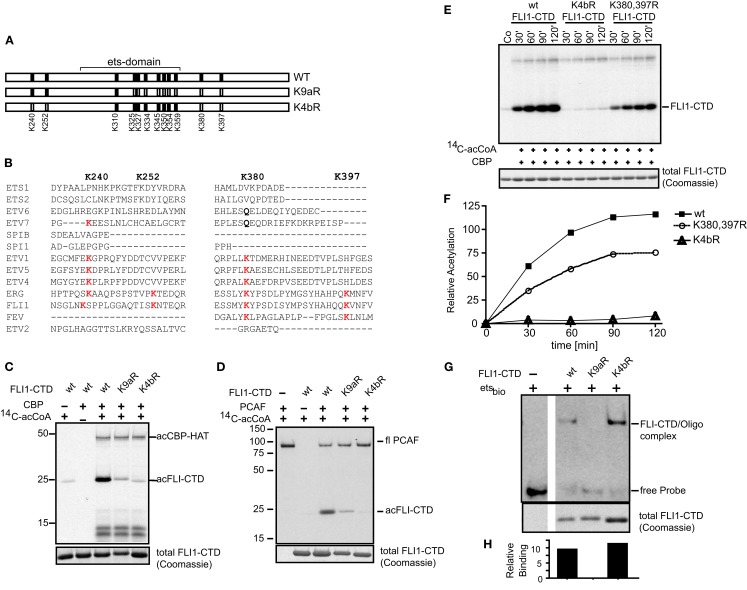
**Mutagenesis confirms K240, K252, and K380 as major acetylation sites**. **(A)** Two lysine to arginine mutants were created of the FLI1-CTD of EWS-FLI1 using site directed mutagenesis. K4bR (K240,252,380,397R) and K9aR (K325,327,334,345,350,354,359,380, 397R). **(B)** Protein sequence alignment of different ets family members at relevant lysine positions. Protein sequences of ETS1 (P14921), ETS2 (P15036), ETV6 (P41212), ETV7 (Q9Y603), SPIB (Q01892), SPI1 (P17947), ETV1 (P50549), ETV5 (P41161), ETV4 (P43268), ERG (P11308), FLI1 (Q01543), FEV (Q99581), and ETV2 (O00321) were aligned using UniProt, to reveal conservation of Lysine positions K240, K252, K380, and K397. **(C,D)** Acetylation mutants were *in vitro* acetylated by CBP **(C)** or PCAF **(D)**. **(E)** Kinetic of CBP *in vitro* acetylation of K4bR and K380, 397R double mutant proteins. **(F)** Densitometric analysis of **(E)** (wt, filled squares; K4bR, open triangles; K380,397R double mutant, open circles). For *in vitro* acetylations dried gels were re-hydrated after exposure and stained for Coomassie to show equal loading [lower panels **(B–D**,**F)**]. This experiment was performed only once in this kinetic fashion. **(G)** Non-radioactive EMSA using ets-binding site containing oligonucleotides and mutant recombinant proteins. Fractions of the EMSA samples were analyzed by Coomassie (lower panel). **(H)** Densitometric analysis of images shown in **(G)**. Relative binding = EMSA signal/Coomassie signal.

The ets family of proteins shows modest conservation of lysine, or related glutamine at residues we identified in the MS analysis (Figures 4A,B). We validated the acetylation residues by creating lysine (K) to arginine (R) mutations. These amino acid substitutions are known to cause relatively conserved structural changes but prevent acetylation. K4bR is a FLI-CTD protein that has the four lysine residues 240, 252, 380, and 397 mutated to arginine (Figure 4A). These lysine residues are located outside the ets domain. Lysine 397, while not specifically detected by MS analysis, was included because it contributed to the effect of K380 acetylation in full-length FLI1 (Asano et al., 2007). The mutant K9aR has 9 out of 12 lysines in the FLI1-CTD mutated to arginine, which includes the ets domain lysines 325, 327, 334, 345, 350, 354, 359 (except 310), and the two lysines 380 and 397 outside the ets domain (refer to Figures 3A and 4A for positions). K4bR showed abolished CBP as well as PCAF mediated acetylation, however K9aR (with K240 and K252) had a small amount of residual acetylation (Figures 4C,D). We also mutated individual acetylation sites and paired lysine positions. While single mutants did not have an effect on the overall acetylation signal (data not shown), subsequent *in vitro* acetylation studies showed reduced CBP acetylation kinetics of the double mutant K380, 397R (Figures 4E,F). This kinetic assay confirmed that the K4bR mutant was unable to be acetylated. Similarly, PCAF acetylation of the double mutant was reduced (data not shown). Thus, we concluded that K240, K252, and K380 represent the three main acetylation sites of the C-terminal FLI1 domain of the fusion protein, with K397 potentially serving a cooperative function.

The DNA binding ability of a protein depends on the structural integrity of its DNA binding domain. EMSA with the K4bR and K9aR proteins were performed in order to determine if the multiple mutations would disrupt DNA binding. The mutant K9aR lost its ability to bind to an ets-binding site containing oligonucleotide in a non-radioactive EMSA, while K4bR retained similar DNA binding to that of the non-acetylated wild-type protein (Figures 4G,H). The K4bR mutant, at baseline, has approximately the same DNA binding activity as the non-acetylated wild-type protein when controlled for the amount of protein in the assay (Figure 4H). This implies that in K4bR structural integrity is maintained, in contrast to the mutant K9aR.

### ES cells express histone acetyltransferases

**Figure 5:**
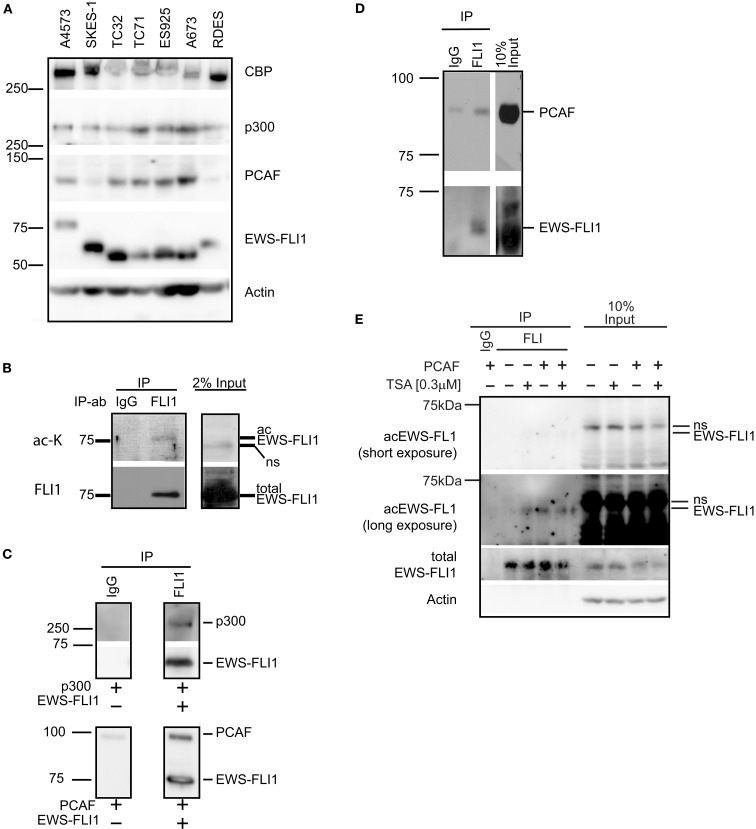
**Histone acetyltransferases are expressed in ES cells and directly interact with EWS-FLI1**. **(A)** Whole cell lysates of various ES cells were tested for expression of CBP, p300, and PCAF protein by western blot. **(B)** EWS-FLI1 was immunoprecipitated from A4573 cells with a FLI1 antibody to detect acetylation by western blot using a pan-α-acetyl lysine. **(C)** Recombinant EWS-FLI1 directly binds to recombinant p300 (upper panel) and recombinant PCAF (lower panel). One microgram of each recombinant protein was used. **(D)** TC32 cells were transfected with PCAF expression plasmid. After 24 h lysates were subjected to immunoprecipitation with FLI1 antibody to show that the EWS-FLI1 PCAF complex also occurs in ES cells. **(E)** TC32 cells were transfected with PCAF followed by treatment with 0.3 μM TSA for 16 h. Whole cell lysates were immunoprecipitated with an anti-FLI1 antibody. A pool of site-specific anti-acetyl-EWS-FLI1 antibodies was used to detect acetyl-EWS-FLI1 (antibodies to K240Ac, K252Ac, K380Ac, K397Ac. For antibody generation, refer to see Experimental Procedures). The same membrane was stripped and reprobed with α-FLI1 and α-actin.

Histone acetyltransferases are important co-factors for transcription; as such they are also implicated in the transcription of oncogenic and tumor suppressing transcription factors [e.g., c-myc (Patel et al., 2004) and p53 (Di Stefano et al., 2005)]. To investigate the potential biological importance of different HATs in ES cells we first determined which HATs are expressed in ES cells (Figure 5A). We determined that all seven ES cell lines tested expressed both p300 and CBP. Intriguingly, PCAF was expressed by all type I and type III EWS-FLI1 expressing cell lines, while EWS-FLI1 type II cell lines, SKES-1 and RDES, only weakly express PCAF. While we were not able to detect acetylated EWS-FLI1 in total cell lysate, when EWS-FLI1 was enriched by immunoprecipitation from A4573 cell lysate, we observed that EWS-FLI1 type III is acetylated *intracellular* using a general acetyl-lysine antibody (Figure 5B). Since this was a pan-acetyl-lysine antibody, we therefore assumed that the detection of acetyl EWS-FLI1 was reduced due to low antibody affinity. Therefore we generated novel acetyl-EWS-FLI1 specific antibodies for following applications.

### P300 and PCAF form a complex with EWS-FLI1

**Figure 6:**
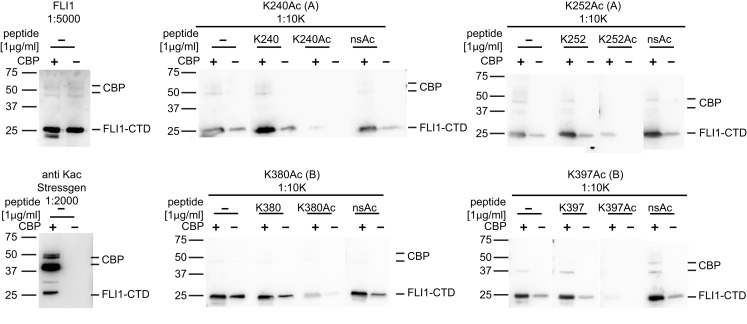
**Specificity of EWS-FLI1 acetylation site-specific antibodies**. FLI1-CTD protein was *in vitro* acetylated using non-radioactive acetyl-CoA and CBP (+) or non-radioactive acetyl-CoA without CBP (−). Aliquots of the same samples were separated on SDS PAGE and strips were incubated with antibodies as indicated. For peptide competition antibodies were incubated with 1 μg/ml of K240Ac, K252Ac, and K380Ac or 5 μg/ml K395Ac peptides.

Since ES cells express all major HATs, interactions between EWS-FLI1 and the different HATs were investigated. Immunoprecipitation experiments with purified recombinant EWS-FLI1 and p300 PCAF proteins demonstrated that EWS-FLI1 complexes with both HATs (Figure 5C). In cell based experiments, we showed that endogenous EWS-FLI1 was only able to co-precipitate PCAF when it was exogenously expressed in TC32 cells (Figure 5D). When endogenous EWS-FLI1 was immunoprecipitated from TC32, no acetylation was detectable. However, when TC32 cells were either co-transfected with PCAF or treated with the HDI TSA, EWS-FLI1 acetylation could be detected in immunoprecipitated protein using a pool of acetyl-EWS-FLI1 specific antibodies (Figure 5E). These acetylation specific antibodies are novel reagents that were prepared by immunizing rabbits with acetylated peptides (see Experimental Procedures). Specificity of these antibodies was confirmed with competition studies using acetylated peptide (Figure 6). Thus, acetylated EWS-FLI1 should be detectable in ES cells with appropriately sensitive reagents.

### Site-specific antibodies confirm intracellular acetylation leading to enhanced EWS-FLI1 transcription

**Figure 7:**
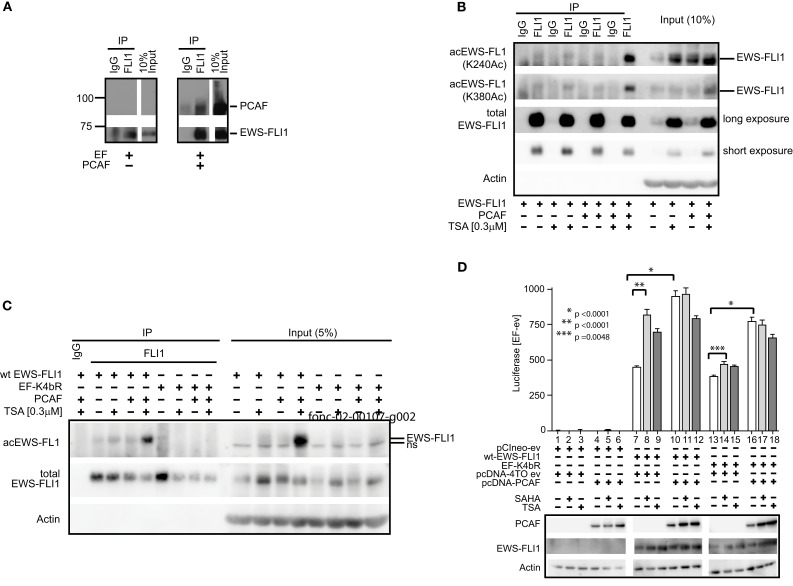
**EWS-FLI1 acetylation increases upon HDI treatment and co-expression of PCAF**. **(A)** Cos 7 cells were co-transfected with EWS-FLI1 type I and PCAF-expression plasmid or empty vectors to show that EWS-FLI1 interacts with PCAF in Cos 7 cells. Thirty hours post transfection cells were lysed and lysate was subjected to immunoprecipitation with α-FLI1 or IgG antibodies. Western blots were probed with α-FLI1 and α-PCAF antibodies. **(B)** Cos 7 cells were transfected with wt EWS-FLI1, with or without PCAF. Acetylated EWS-FLI1 was detected by single site-specific antibodies K240Ac and K380Ac. **(C)** Cos 7 cells were transfected with wt EWS-FLI1 or EF-K4bR constructs, with or without PCAF. **(D)** Cos 7 cells were transfected using pCIneo empty vector, wt-EWS-FLI1, or its acetylation mutant EF-K4bR in combination with pcDNA-4TO empty vector or pcDNA4TO-PCAF and the NR0B1 reporter construct. The next day cells were treated with SAHA (2 μM) or TSA (0.3 μM) for 8 h (control: untreated) and then subjected to luciferase and western blot analysis. Luciferase activity was expressed as luciferase activity (sample) – luciferase activity (empty vector control) containing both empty vectors (pcDNA4TO and pciNeo). Bar graphs result from triplicate transfection and duplicate luciferase measurement. Two-tailed *t*-test was performed using GraphPad Prism version 4.00.

EWS-FLI1 (type 1), and PCAF were co-transfected into Cos7 cells and this complex was confirmed by co-immunoprecipitation (Figure 7A). Acetylation was barely detectable under basal conditions of expressed EWS-FLI1. However, when cells were co-transfected with PCAF and treated with TSA, acetylation increased. The combination of TSA treatment and PCAF expression significantly enhanced our ability to observe EWS-FLI1 acetylation at residues K240 and K380 with site-specific antibodies (Figure 7B). In contrast, the full-length EF-K4bR mutant was not acetylated with basal expression, nor could it be acetylated with optimal acetylation conditions using expressed PCAF and TSA treatment (Figure 7C). Therefore, we used the EF-K4bR mutant (K240,252,380,397R) to evaluate transcriptional activation. Wild-type EWS-FLI1 was transfected into Cos7 cells and treatment with either SAHA or TSA increased transcriptional activity (Figure 7D, comparing lanes 8 or 9 to lane 7).

PCAF expression also increased EWS-FLI1 transcriptional activity (Figure 7D, comparing lanes 10–7). The EF-K4bR mutant had slightly less activity than wild-type EF, however unlike the wild-type protein, the luciferase activity did not significantly increase with either SAHA or TSA (Figure 7D comparing lanes 14 or 15 to lane 13). When the EF-K4bR mutant was co-expressed with PCAF, promoter activity increased, and like wild-type EF, no additional increase was seen with the HDI. Although less active than EWS-FLI1, the EF-K4bR was still responsive to PCAF stimulation, but it was insensitive to treatment with HDIs. This supports a role for these acetylation sites in EWS-FLI1 activated transcription. That PCAF also increased promoter activity even when EWS-FLI1 cannot be acetylated at the four lysine sites (240, 252, 380, 397) points to additional PCAF acetylation sites within the protein.

## Discussion

The direct functional modulation of transcription factors by acetylation is an area of significant research. Our initial hypothesis asked whether EWS-FLI1 activity was modulated by acetylation. We showed that co-expression of PCAF or p300 increased the promoter activation by EWS-FLI1. Following this, we determined that acetylation of the FLI-CTD increased DNA binding affinity, as one explanation of this increased promoter activity. We then sought to identify the specific residues of EWS-FLI1 that are acetylated, first using *in vitro* acetylation reactions followed by MS. The FLI-CTD fragment led us to identify K240, K252, and K380 as probable sites for acetylation. Mutagenesis followed by acetylation assays confirmed these sites. We then showed that ES cells expressed and EWS-FLI1 bound to the various acetylases, but required exogenous expression for this co-precipitation. Finally we confirmed K240 and K380 as sites of acetylation in co-transfected COS cells and that acetylation at these sites increased EWS-FLI1 driven promoter activation. Overall, our data supports a role for acetylation in EWS-FLI1 mediated transcriptional regulation. However, challenges in identifying an acetylation mark upon full-length EWS-FLI1 using *in vitro* acetylation along with an absence of detectable acetylation in ES cells prevents a clear acceptance of our hypothesis.

Transcription factors belonging to the *ets* family are regulated by acetylation (Goel and Janknecht, 2003; Bai et al., 2005). Pu.1 is a substrate for p300 with the three major acetylation sites residing within the ets domain. The transcriptional activity of Pu.1 was increased by p300 interaction as well as treatment with the HDI, TSA (Bai et al., 2005). ER81 interacts with, and is a substrate for, both p300 and PCAF. In contrast to Pu.1 acetylation takes place outside the ets domain at the N-terminal of Pu.1. However, both HATs also increase transcriptional activation and DNA binding of ER81 (Goel and Janknecht, 2003). Wild-type FLI1 is acetylated by PCAF upon stimulation with TGFβ in dermal fibroblasts (Asano et al., 2007). The acetylation of FLI1 at lysine 380 abrogates the repressor function of Fli1 and shortens its half-life, thus reducing its DNA binding and transcriptional activity.

We have identified multiple relevant acetylation sites in the C-terminal FLI1 domain of the fusion protein including lysine residues K240, K252, and K380 (referring to the positioning within the wt FLI1 protein). This conclusion is based upon a combination of MS, mutagenesis and novel site-specific acetylation antibodies. Our experimental results are in agreement with observations for the ETS proteins Pu.1 and ER81. An increase of transcription and DNA binding upon co-expression of HATs has been observed for both proteins in response to HDI treatment (Goel and Janknecht, 2003; Bai et al., 2005). Additionally ER81 becomes stabilized upon acetylation.

The key question is why these acetylation marks are not observed in ES cell lines. In our experiments, detection of acetylated EWS-FLI1 required significant amplification through either protein over-expression or drug treatment. Thus, our findings pose the question: is EWS-FLI1 actually regulated by acetylation and if so, why is acetylated EWS-FLI1 not more abundant? One possibility is that the full-length fusion protein alters the ability of acetylases to bind and in fact, EWS-FLI1, unlike wild-type FLI1, may not be regulated by acetylation. Since EWS-FLI1 is an intrinsically disordered protein, its solvent exposure in cells, thus its exposure to enzymatic alteration is likely different from one of its domains (Erkizan et al., 2010). This escape from regulation may be another manifestation that leads to oncogenesis.

An alternative explanation considers that most cells do not tolerate (over-) expression of exogenous EWS-FLI1 (Tanaka et al., 1997). Situations that are permissive of EWS-FLI1 expression include secondary mutations in growth and cell cycle controlling genes like p53 or p16 (e.g., Deneen and Denny, 2001). Acetylation increases transcription and DNA binding of EWS-FLI1, therefore, the additional potency might subdue the cells. It is however also possible that the inability to clearly detect acetylation of full-length EWS-FL1 is due to the limited sensitivity of the specific antibodies directed against acetylated forms of the protein. For example, in the case of many other proteins, including p53, it has been historically difficult to accurately estimate the ratio between acetylated and non-acetylated forms.

The balance of the effects of EWS-FLI1 acetylation, in contrast to the general effects HDI have upon ES cells is experimentally challenging. The acetylation of EWS-FLI1 under native conditions is low, but present, as shown by novel acetyl-specific antibodies. The importance of the single sites and their biologic significance remains cryptic despite the increase of acetylation at all four individual lysines by PCAF. Similar to the oncogenes p53 and c-Myc the sites might have different relevance for a differential regulation of EWS-FLI1. P53 is acetylated at two different sites which differentially regulate its binding to DNA and its ability to induce an apoptotic response in lung carcinoma cells (Knights et al., 2006). c-Myc, is differentially regulated by hGCN5/PCAF (Patel et al., 2004), and p300/CBP (Faiola et al., 2005), resulting in altered protein stability (Faiola et al., 2005).

Acetylation of K380 and K397 decrease the stability of full-length wild-type FLI1, resulting in FLI1 protein degradation (Asano et al., 2007). We had initially hypothesized acetylation of EWS-FLI1 might be relevant for its protein stability as well, similar to previous publications (Sakimura et al., 2005), yet multiple repeated experiments did not demonstrate altered stability upon increased acetylation. Our specific experiments addressing EWS-FLI1 half-life were confounded by ES cell sensitivity to agents like cycloheximide or proteasomal inhibitors, traditionally used to block *de novo*-protein synthesis or protein degradation, did not allow for the necessary incubation times to complete experiments addressing protein stability.

An increasing number of clinical trials are investigating HDI in different tumors emphasizing their potential as omnipotent anticancer agents. However, as more and more non-histone proteins are revealed as acetylated, consideration of potential effects toward transcription factors like c-Myc (Vervoorts et al., 2003; Faiola et al., 2005; Zhang et al., 2005), or p53 (Knights et al., 2006) have to be considered. ES cell lines and other pediatric tumor cell lines are relatively sensitive to HDIs (Furchert et al., 2007; Sonnemann et al., 2007). With the number of non-histone targets for acetylation increasing (Choudhary et al., 2009), consideration of HDI effects upon non-histone proteins should be investigated in terms of overall toxicity toward tumor cells. A key caveat to our work is our limited scope of promoter evaluation, such that future experiments might find differential effects of acetylation based upon the specific EWS-FLI1 regulated promoters (or gene expression) that are studied. As HDI will be used in Phase II trials of ES (and other pediatric tumors), we investigated HDI as modulators of EWS-FLI1.

Our observations of increased transcriptional and DNA binding activity seems a relative contraindication for application of HDI for ES therapy. However, ES cells undergo apoptosis despite (or even because of) the increased transcriptional activity of EWS-FLI1. While the role acetylation plays in EWS-FLI1 regulation is not yet fully evaluated, we have developed useful tools (acetylation specific EWS-FLI1 antibodies). It would be prudent to incorporate more clinically relevant models to investigate a potential clinical correlation of the EWS-FLI1 acetylation status with therapy responsiveness. Thus, well-informed clinical trials with HDI in ES should proceed cautiously based upon overall anti-tumor effects. As with other targeted therapies, nuances of the target may be critical to understand in order to derive the greatest clinical benefits.

## Conflict of Interest Statement

The authors declare that the research was conducted in the absence of any commercial or financial relationships that could be construed as a potential conflict of interest.

## Abbreviations

comp, competitor; ES, Ewing sarcoma; HAT, histone acetyltransferase; HDAC, histone deacetylase; HDI, histone deacetylase inhibitor(s); MS, mass spectrometry; SAHA, suberoylanilide hydroxamic acid, also known as vorinostat, marketed as Zolinza; TSA, trichostatin A.
